# Ionic Surface Active Compounds in Atmospheric Aerosols

**DOI:** 10.1100/tsw.2002.188

**Published:** 2002-04-27

**Authors:** Jariya Sukhapan, Peter Brimblecombe

**Affiliations:** School of Environmental Sciences, University of East Anglia, Norwich NR4 7TJ, UK

**Keywords:** methylene blue active substances, surfactants, atmospheric aerosol

## Abstract

Surfactants in the atmosphere have several potential roles in atmospheric chemistry. They can form films on aqueous surfaces, which lowers the surface tension and possibly delays water evaporation and gaseous transportation across the aqueous interface. They can also increase the solubility of organic compounds in the aqueous phase. Recently, the decrease of surface tension in cloud growing droplets has been suggested as relevant to increases in the number of droplets of smaller size, potentially enhancing cloud albedo. Natural surfactants in the lung aid gas transfer and influence the dissolution rate of aerosol particles, so surfactants in atmospheric aerosols, once inhaled, may interact with pulmonary surfactants. Ambient aerosols were collected from the edge of Norwich, a small city in a largely agricultural region of England, and analysed for surfactants. Methylene blue, a conventional dye for detecting anionic surfactants, has been used as a colorimetric agent. The concentration of surfactants expressed as methylene blue active substances (MBAS) is in the range of 6–170 pmol m(air). A negative correlation with chloride aerosol indicates that these surfactants are probably not the well-known surfactants derived from marine spray. A more positive correlation with aerosol nitrate and gaseous NO_x_ supports an association with more polluted inland air masses. The surfactants found in aerosols seem to be relatively strong acids, compared with weaker acids such as the long-chain carboxylic acids previously proposed as atmospheric surfactants. Surfactants from the oxidation of organic materials (perhaps vegetation- or soil-derived) seem a likely source of these substances in the atmosphere.

## INTRODUCTION

Surface active compounds are commonly found in the environment, resulting from both human activities and natural processes. These compounds are best known as detergents in surface waters and the foam producing layers on the ocean surface[1,2,3,4,5]. Although surface active substances have not been as widely studied in the atmosphere, an awareness of their presence stretches back two decades[1,2]. Macromolecules such as humic acids, proteins, and protein breakdown products are typical of naturally occurring surfactants[1,2,3,4,5,6,7]. Derivatives of aliphatic hydrocarbons (represented by carbonyls, esters, ketones, alcohols, and amines) have been seen as the typical surface active compounds in the atmosphere, with a general view that atmospheric surfactants are likely to be long-chain fatty acids[1]. Observational support of this derives from the work of Seidl[2,3].

Surface active compounds are seen as potentially important in environmental chemistry because of their ability to alter the movement of materials across aqueous interfaces and to solubilize compounds in aqueous systems[8,9,10,11,12]. In the marine environment surfactants at the sea surface play a role in the recycling and long-range transport of pollutants via marine aerosols[4,5]. Heavy metals — pollutants from crustal and urban sources — enriched on the sea surface were found to interact with the surface-active organic matter and become transferred into marine spray[4].

The early reports of surface active materials in rain water and atmospheric aerosols found that their concentrations were too low to have any effect upon the cloud physical process[2] at high dilution. On more-concentrated, smaller, or growing droplets the situation may be different. In such situations a decrease in surface tension of cloud droplets could lead to an increase among the smaller sizes of the droplet population. The lifetime of clouds could be prolonged, resulting in an overall enhancement of cloud albedo and an associated cooling effect in the atmosphere[13,14,15].

In the indoor atmosphere, the surfactants derived from cleaning agents have been argued as an agent promoting sick-building syndrome[16,17]. They cause irritation and dry eyes by reducing the surface tension of the tear film. Alkyl benzene sulfonates and fatty acid salts, two important surfactants used in cleaning clothes, have been found in the indoor dust[18,19]. It has been argued that respirable particles contaminated with surfactants can destabilise the mucous membrane of the respiratory system leading to allergies and asthma[16,17].

This paper reports a series of measurements of the surfactant concentration in aerosols at a semirural site in the U.K. using the methylene blue method.

## METHOD

Aerosol samples were collected without size discrimination from the roof of the School of Environmental Sciences building at the University of East Anglia, Norwich, during four campaigns over the period from December 2000 to August 2001. The campaigns represented semicontinuous monitoring over 2- to 3-week periods chosen to represent different seasons. The site was some 40 m above sea level and 25 km from the North Sea coast. A mass flow control aerosol sampler operated at a flow rate of approximately 1 $m^3$ $min^{-1}$ collecting samples over a 24 hour period. The filters were weighed before and after sampling at a constant relative humidity (50% RH ) to determine the aerosol mass collected.

The first five samples were collected on cellulose acetate filters, but these gave a significant positive blank value (12 pmol $m^{-3}$ air) in the methylene blue analysis. Subsequently glass fibre filters prefired at 350°C for 3 h were used because they gave lower positive background. With glass fibre filters, the background was equivalent to a blank correction in the measurements of about 6 pmol $m^{-3}$ air.

The water soluble components of atmospheric aerosol were extracted by ultrasonication and filtration. The analysis for anionic surfactants utilised a methylene blue method modified from that of Mathijis[20] and Srinivasarao et al.[21], which has often been adopted for marine surface water and marine aerosols[4]. The aqueous solution containing anionic surfactants was mixed with an alkaline solution of methylene blue, then the complex between surfactants and methylene blue was extracted with chloroform. The chloroform extract was back-extracted into an acidic solution of methylene blue. Interferents such as nitrates, sulfates, and carboxylates are reduced by this second extraction. The methylene blue bound surfactants were determined spectrophotometrically in the chloroform extract.

Methylene blue is one of a number of colorimetric reagents for anionic surfactants. It is the most commonly used because the two-step extraction reduces interferences. Known interferents include anions such as chloride (important in marine samples), but in our work the chloride concentrations were such that the signal would have been less than our typical analytical errors. The method is rapid and simple, with the advantage of detecting a wide range of surfactant compounds; e.g., linear alkyl or moderately sized aromatic (e.g., naphthalene sulfonate) or cyclic compounds. Because the method detects groups of compounds, the results are best expressed as the concentration of methylene blue active substances (MBAS). This concentration was calculated by comparison of standards containing sodium dodecyl sulfate, which has a known molecular weight. The lower limit of detection of MBAS was 2 pmol $m^{-3}$ air. A limited range of measurements was made for cationic surfactants using the disulfine blue method modified from that of Waters et al.[22,23]. By comparative calculation with zephiramine, the cationic surfactant concentration was expressed as the concentration of disulfine blue active substances. The lower limit of detection was similar to that for the anionic determination.

The inorganic contents (chloride, sulfate, and nitrate) in the water soluble fraction were determined by using DIONEX 4000i ion chromatography. The column used was an AS14 separator column protected by an AG14 guard column with a 3.5 mM $Na_2CO_3$ and 1.0 mM $NaHCO_3$ eluent. The detection limit was 1 nmol $m^{-3}$.

## MEASUREMENTS AND DISCUSSION

Methylene blue active substances indicative of anionic surfactants were detected in all the samples, ranging from 6 to 170 pmol $m^{-3}$. Without knowledge of the molecular weight of the surfactants it is not possible to express concentration as a weight; however, for comparison one could follow Oppo et al.[4] and Longwell and Maneice[24], who express the concentration range of surfactants as 2–76 ng $m^{-3}$ air by using sodium dioctyl sulfosuccinate (MW 444.6) as the reference compound. The breakdown of data into summer, winter, and spring campaigns is given in Table 1, which lists the concentration of MBAS, both in terms of airborne concentration and as a concentration within the aerosol.

The frequency distribution of data did not appear to be normal or log-normal; thus, we took the median as a measure of central tendency and used the Kruskal-Wallis test to show a significant difference between the medians for the three seasonal measurements expressed as pmol $m^{-3}$ in air ($\chi^2 = 17.46$, df = 2, $p < 0.001$). There was no significant difference between seasonal data expressed as a fraction of aerosol mass; i.e., μmol $g^{-1}$ aerosol ($\chi^2 = 2.95$, df = 2, p = 0.229).

It is clear that the concentrations in air are highest in winter. These are similar to the concentrations (range 47–260 pmoles $m^{-3}$) of weakly soluble and insoluble film-forming compounds in atmospheric particles, determined from film balance and tensiometric measurements of Seidl and Hanel[2]. When our concentrations are expressed as a fraction of the

**TABLE 1**
**Range of Methylene Blue Active Substances (MBAS) in Aerosol Samples Collected during Three Seasons**

| Sampling period | No. of samples | MBAS pmol $m^{-3}$ air | | MBAS µmol $g^{-1}$ aerosol | |
|---|---|---|---|---|---|
| | | range | median | range | median |
| Winter [December-February] | 28 | 16–168 | 71 | 0.28–3.07 | 1.2 |
| Spring [May] | 15 | 6–80 | 34 | 0.28–2.33 | 0.9 |
| Summer [July-August] | 28 | 13–82 | 43 | 0.31–2.69 | 1.15 |

*Note:* The error in the measurements in pmol $m^{-3}$(air) and µmol $g^{-1}$(aerosol) were 8 and 5%, respectively.

aerosol mass, they are only slightly higher in winter than other seasons because the aerosol is generally more abundant at the Norwich site in winter.

The concentrations of cationic surfactants were measured as 4 pmol $m^{-3}$ air collected over 48-h periods in November 2000. This result suggested that the concentrations of positively charged surfactants at this site were typically very low. This value is only twice our estimate of the detection limit, so this monitoring was not continued.

Surfactant concentrations have been expressed in picomoles per cubic metres of air, which indicates very low airborne concentrations; however, these concentrations need to be considered relative to the amount of water in the atmosphere. Facchini et al.[13] suggest surfactant concentrations in the range $10^{-4}$–$10^{-2}$ M are sufficient to affect the rate of droplet growth. In order to reach such concentrations inside aerosol droplets, the water content has to be in the range 0.01–1 mg/$m^3$ (air) if surfactant concentration was set at 100 pmoles $m^{-3}$. Amounts of liquid water as low as this are reasonable for growing aerosol particles[13].

In addition to the effects on surface tension, aerosol surfactants could solubilize normally insoluble organic compounds of low polarity into aqueous solutions associated with aerosols. Solubilization depends on the critical micelle concentration (CMC) of the surfactant under consideration. This concentration is not known for the MBAS detected here. In a trial calculation we presumed the MBAS might be similar to cholic acid, a natural steroid-like molecule that acts as a surfactant. This compound is a possible choice for atmospheric surfactants because sterols have been found in the atmosphere in pmol $m^{-3}$ concentration range[25]. Cholic acid has a CMC of 4 mM[26], which suggests that it would be able to solubilize organic compounds under conditions of relatively low atmospheric liquid water content.

Fig. 1 shows MBAS concentration as a function of aerosol loading. The lack of significant correlation (typically explaining only 15% of the variance) suggests that surfactants on aerosols are not simply related to the amount of aerosols available. It makes it likely that they have a more distinctive source or sources. The very high aerosol load denoted on the graph by an arrow arose on a day with perceptible local pollution from domestic fireplaces; however, a lack of a corresponding increase in MBAS implies that fresh domestic smoke did not represent an important source of the surfactant.

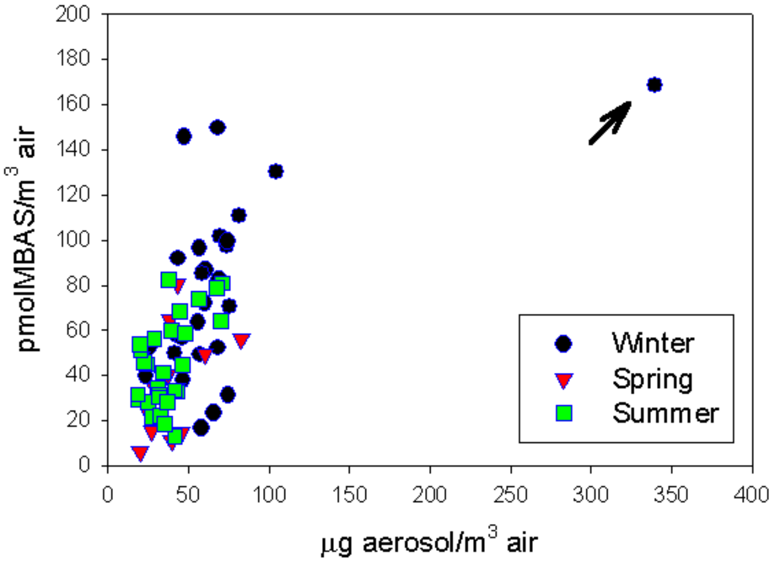

**FIGURE 1.** Methylene blue active substances (MBAS) concentration in the atmosphere as a function of aerosol loading. Arrow indicates day of perceptible local pollution from domestic fireplaces.

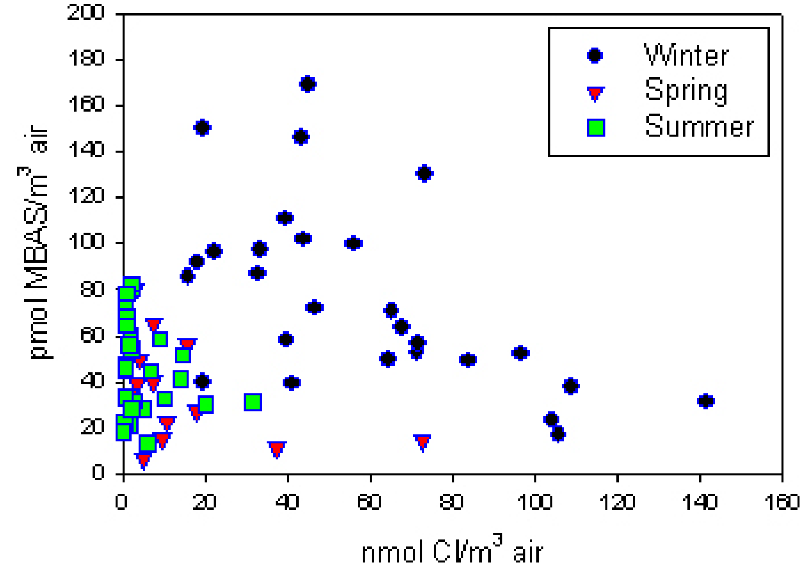

**FIGURE 2.** MBAS concentration in the atmosphere as a function of aerosol chloride content.

Some general ideas for the source of MBAS in aerosols can be derived from the presence of inorganic anions. Overall, the aerosol surfactant concentration did not show a strong correlation with the chloride content (Fig. 2); however, in winter it was a noticeably inverse correlation suggesting the MBAS found in this study did not principally derive from the familiar surfactants of marine aerosols. The hibernal (winter) correlation coefficient ($r_s$) between MBAS concentration and chloride content was –0.54 with n = 27 and p = 0.003. We have chosen the Spearman correlation coefficient because it is less sensitive to the distribution of the data. A positive correlation is found with nitrate (Fig. 3A) and sulfate (Fig. 3B) content of the aerosols.

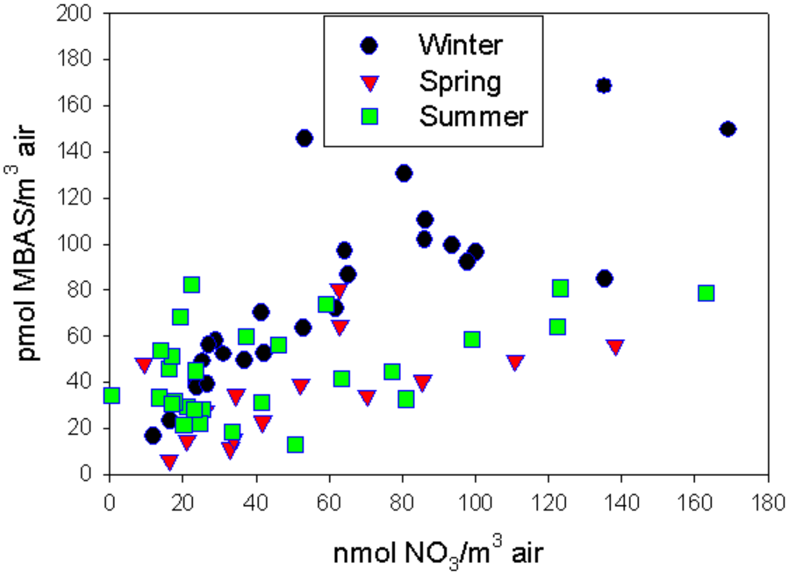

**FIGURE 3A**

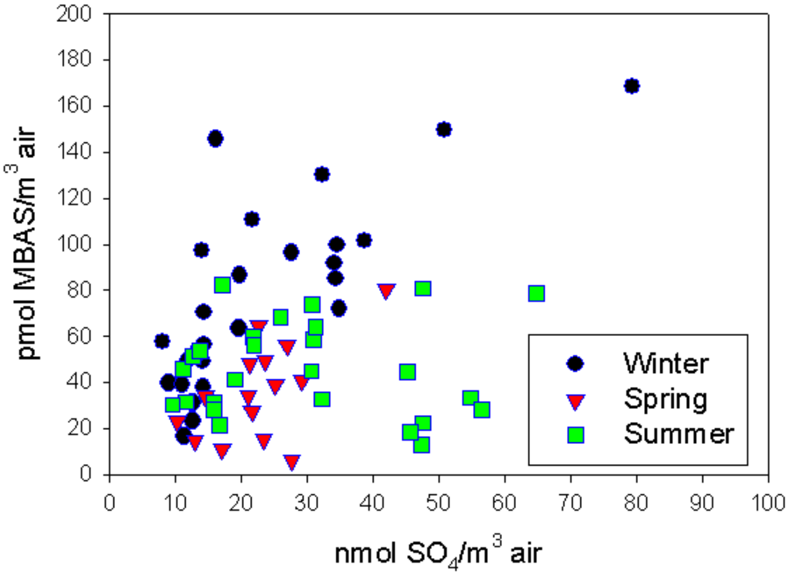

**FIGURE 3B**

**FIGURE 3.** (A) MBAS concentration in the atmosphere as a function of aerosol nitrate content. (B) The MBAS concentration in the atmosphere as a function of aerosol sulfate content.

Once again the relationship is clearer for the winter samples ($r_s = 0.89$, $n = 27$, $p < 0.001$ with nitrate, and $r_s = 0.79$, $n = 27$, $p < 0.001$ with sulfate) partly because of the broader concentration ranges. A reasonable correlation ($r_s = 0.67$, $n = 66$, $p < 0.001$) is also found between the MBAS concentration and $NO_x$ concentration (Fig. 4). The values here were taken from the automated rural site at Wicken Fen, some 80 km to the west[27]. There was also a relationship with $NO_x$ within the nearby city of Norwich ($r_s = 0.71$, $n = 70$, $p < 0.001$), but it is likely that the rural site

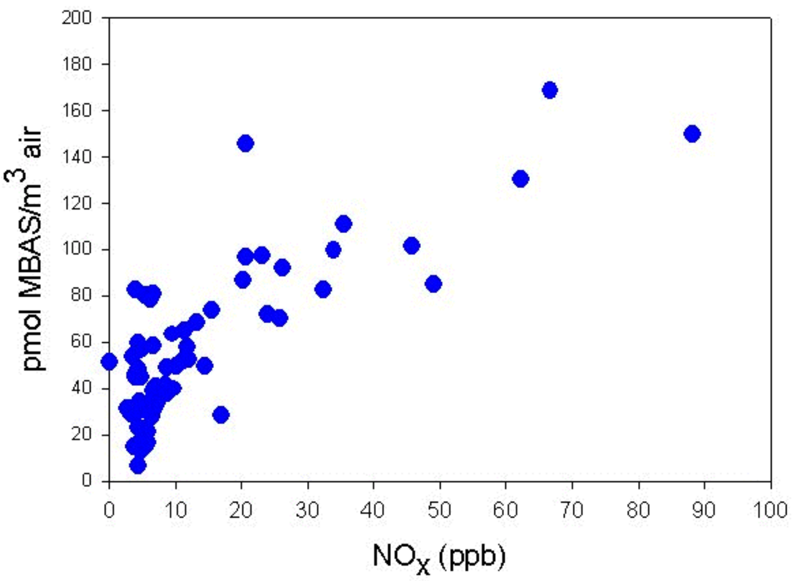

**FIGURE 4.** The MBAS concentration in the atmosphere as a function of $NO_x$ concentration.

will be more representative of background $NO_x$ experienced by the air masses. There was an inverse relationship between MBAS concentration and ozone, which offered little support for the substances being derived from highly oxidising air masses.

Although we examined 24-h back trajectories for each sampling day as constructed by the Hybrid Single-Particle Lagrangian Integrated Trajectory program[28], no clearer indications of surfactant source emerged with regard to wind direction or the nature of the trajectory. We also looked at *Lamb Weather Types* (from Climatic Research Unit, UEA, Norwich, U.K.[29]), but again these offered no further clarity.

## CANDIDATE COMPOUNDS

It is often assumed that atmospheric surfactants are long-chain carboxylic acids such as stearic and palmitic acids. Observational evidence of film formation in rainwater and atmospheric particles from Seidl would not discount these simple organic acids[2]; however, stearic and palmitic acids are very weak and do not give positive results with the methylene blue method under acidic conditions. The atmospheric materials detected in this work were active with methylene blue even under acidic conditions, which suggests that they are likely to be stronger acids. This finding is of some importance as the atmospheric aerosol is typically acidic (pH 3–5), and more-acidic surfactants should be more effective at changing the physical properties of liquid water in the atmosphere than those derived from weaker carboxylic acids.

In addition, simple carboxylic acids are rather ineffective at solubilizing materials. Caprylic acid (as the sodium n-octanoate), for example, has a CMC of 351 mM[26]. Sulfonates and some steroid-like compounds, such as cholic acid, are more effective (CMC in the range of 2 to 7 mM for various cholic acid derivatives). There are also sulfonated sterols such as the taurocholic acid, which has a pK as low as 2.

We have tested a range of potential source materials — leaf fragments, road dust, and charred organic material — all of which had exceedingly low MBAS (0.002–0.007 μmol $g^{-1}$ material or dust) compared with aerosols, so these do not seem likely sources. We also considered that the surfactant detected might be due to localised detergent emissions, such as from laundries.

Although plausible, such a possibility would hardly explain the correlations with chloride and nitrate shown in Figs. 2 and 3. Preliminary experiments suggest that humic substances can be weakly active towards methylene blue. Humic materials are typically macropolycarboxylic acids, so methylene blue activity is not surprising. The decomposition of humic acid solutions in light might generate greater activity[30]. Sterols emitted from biomass processes are another potential source. Steroid skeletons could gain more polarity via the oxidation in the atmosphere and become effective surfactants during long-range transport[25,31,32].

## CONCLUSIONS

Anionic surface active compounds (detected as MBAS) have been found in the 100 pmol/cubic metre concentration range. By contrast, barely detectable concentrations of cationic surfactants were found. Since oxidation processes are so characteristic of the atmosphere, anionic surfactants typified by carbonyls (probably acids) seem likely. Despite the low airborne amounts of MBAS, aqueous concentrations could be high enough to change surface tension and form micelles in the limited amounts of water associated with atmospheric aerosols.

The MBAS showed a negative correlation with winter aerosol chloride suggesting that it did not come from the sea. There were positive correlations with aerosol nitrate and $NO_x$, suggesting that the potential surfactants were related to air that originated over land masses, which is often polluted in western Europe. The exact origin of this relatively strong acid surfactant is unclear, but it may be derived from atmospheric transformations of organic materials, possibly organic pollutants or natural precursors such as humic materials or sterols.

## ACKNOWLEDGEMENTS

The study has been supported financially by the Royal Thai Government, Thailand and the U.K. Natural Environment and Research Council's ASURE program (GST/02/1895).

## BIOSKETCHES

**Jariya Sukhapan** was born in Bangkok, Thailand. She gained her B.Sc. and M.Sc. degrees in Biochemistry from Chulalongkorn University and Mahidol University, respectively. Her interest in atmospheric chemistry has resulted in further studies at the University of East Anglia, where she is earning a Ph.D. degree in atmospheric sciences. This work derives from work undertaken as part of this course.

**Peter Brimblecombe** is Professor in Atmospheric Chemistry at the School of Environmental Sciences, University of East Anglia. He holds a Ph.D. in the aqueous chemistry of sulphur. Dr. Brimblecombe was appointed senior editor of *Atmospheric Environment* in 1990. Although retaining an interest in atmospheric sulphur chemistry, he is currently concerned with the thermodynamics of the concentrated aqueous aerosol. This work has more recently focussed on water-soluble organic substances.
